# Epidemiological investigation of a case of malaria in a non-endemic area, Campo de Gibraltar, Cadiz, Spain, January 2022

**DOI:** 10.2807/1560-7917.ES.2022.27.46.2200786

**Published:** 2022-11-17

**Authors:** Boris Verona Mesia, Nuria López-Ruiz, Enric Duran-Pla

**Affiliations:** 1 Public Health, Epidemiology and Health Surveillance Unit Sanitary Management Areas Campo de Gibraltar East and West, Algeciras, Spain; 2 Surveillance and Occupational Health Service, General Directorate of Public Health and Pharmaceutical Regulation, Sevilla, Spain

**Keywords:** Malaria, non-imported case, surveillance

## Abstract

We describe a non-imported malaria case reported in January 2022 in Campo de Gibraltar and the investigations by local public health authorities to identify the transmission mechanism and subsequent measures to prevent local transmission. Vector transmission, parenteral transmission, airport malaria, and imported malaria were ruled out. No clear mechanism of transmission was identified. The most probable cause was a hospital-acquired infection since the case was admitted to hospital at the same time as a case of imported *Plasmodium falciparum* malaria.

Malaria is a disease caused by protozoa of the genus *Plasmodium* and transmitted by the bite of the female *Anopheles *spp*.* It is the most important parasite worldwide, endemic in 85 countries [1]. There are five species of *Plasmodium* capable of causing the disease, with *P. falciparum* being the most serious [2,3]. Since 1964, malaria is considered eradicated in Spain. In January 2022, a case of non-imported malaria was reported in Campo de Gibraltar, in the south of Spain, triggering epidemiological alert. Here we describe the actions carried out in connection with this case, and the conclusions on the management of a non-imported case in a non-endemic country.

## Case presentation

In the second half of January 2022, the Epidemiology Unit of the Campo de Gibraltar East/West Health Management Area (hereafter Epidemiology Unit Area) received the notification of a confirmed case of *P. falciparum* malaria from the preventive medicine service of a private hospital. The patient was a Spanish native in their early 40s, a resident of Campo de Gibraltar, in the province of Cádiz, in Andalusia in the south of Spain. The patient was admitted to hospital 1 month earlier for scheduled surgery and on the day after the surgery, they received a transfusion of packed red blood cells due to moderate anaemia, without other symptoms like fever or haemorrhage. During this admission over 3 days in mid-December, the patient stayed in a private room.

In January 2022, 13 days after the discharge, the patient presented to the emergency room with high fever (40 C°), chills, dysphagia, general malaise, haemoglobin (Hb) of 10.2 gr/dL (norm: 12─15) and platelets of 184,000mm^3^(norm: 150,000─450,000). They were sent home with a diagnosis of a concomitant cold and urinary tract infection. Three days later, the patient returned to the emergency room due to persistent fever plus back pain, Hb was 9.2 gr/dL; the presumed diagnosis remained the same and the patient was not admitted. Another 3 days later, the patient developed weakness, dizziness, and mucocutaneous pallor. The Hb was 6.2 gr/dL, and the patient had thrombocytopenia (83,000mm^3^). It was then decided to admit them to the intensive care unit (ICU) because of haemodynamic deterioration and beginning multiple organ failure with a preliminary diagnosis of haemolytic uraemic syndrome. The main causes of haemolytic uraemic syndrome (*Salmonella, Shigella, Campylobacter and Aeromonas*) were ruled out by negative blood culture tests, a SARS-CoV-2 PCR test was also negative. Four days after admission to the ICU, an antigen test for *P. falciparum* and a thick blood smear were performed and results were positive for both. Treatment with atovaquone 250 mg and proguanil hydrochloride 100 mg, 1 gr every 24 hours, was started and when the patient presented clinical improvement, they were transferred to the main ward, 12 days after admission and discharged 17 days after admission.

## Investigation of possible transmission routes

Malaria cases in non-endemic countries may be due to introduced malaria, imported malaria, airport malaria, congenital or induced (blood transfusions and blood products, post-transplant, parenteral, and healthcare-related) malaria.

## Native *Anopheles* transmission

In Spain, *A. atroparvus* is the historical vector of *P. vivax* and *P. ovale* [4]. Although *A. atroparvus* were considered, to some extent, refractory to tropical strains of *P. falciparum* they can act as vector for *P. falciparum* [5,6]. However, despite *A. atroparvus* presence, cases of autochthonous malaria in Spain are unusual. A fundamental task of the investigation was to rule out whether the infection was acquired by native *A. atroparvus*.

The Epidemiology Unit Area carried out an inspection of the hospital where the case under investigation was admitted. The inspection consisted of checking the hygienic conditions, location and conditions of the room, the area where the hospital centre is located, as well as the laboratory and emergency room for the possible presence of vectors and how and where used materials were disposed. As a result of outbreaks of other vector-borne diseases in previous years in Andalusia, an entomological surveillance programme had been installed [7]. The surveillance is activated during the circulation period of the West Nile virus vector, which runs from March to November every year. For transmission by native *Anopheles* species to occur, the parasite must complete part of its life cycle in the vector, and it may take up to 23 days before the vector can infect a person. Given the short time elapsed since the linked imported malaria case (see below) was admitted in Spain to the identification of the investigated case and additionally that the event took place during the winter period in Spain, when the vector is not circulating, this possibility of transmission is very unlikely, so it was ruled out.

## Imported malaria

As elsewhere in Europe, in Andalusia and Spain malaria cases are mainly imported. In Andalusia 898 confirmed cases have been notified from January 2011 to January 2022, with 99.8% corresponding to imported cases (Table) and especially from Africa.

**Table:** Imported malaria cases by countries of importation, Andalusia, Spain, January 2011 to January 2022 (n = 898)

Originating countries	Cases (n)	%
Mali	301	33.5
Equatorial Guinea	97	10.8
Nigeria	89	9.9
Senegal	79	8.8
Ghana	44	4.9
Ivory Coast	30	3.3
Burkina Faso	28	3.1
Guinea	28	3.1
Guinea-Bissau	24	2.7
The Gambia	17	1.9
Others	161	17.9
Total	898	100.0

Source: Red Alerta Epidemiological Surveillance System. Andalusian Health Service.

Based on the epidemiological interview with the patient, we could rule out that their infection was acquired abroad in a malaria-endemic country, since they had not travelled to endemic areas in the previous 10 years.

## Airport malaria

This type of transmission, caused by imported infected mosquitoes [8], was considered highly unlikely since the nearest airport of Gibraltar does not receive direct flights from malaria-endemic countries. The Gibraltar airport receives flights from the United Kingdom and Malaga and the distance between the airport and the hospital is 8.5 km while local *Anopheles* mosquitoes have a flight range of about 4.5km [2].

## Induced malaria

The patient had no history of using intravenous drugs, tattooing, or body piercing in the last year, thus malaria acquired through such routes was ruled out.

Through the Territorial Health Delegation of Cadiz, the regional transfusion center was requested to provide serological information corresponding to the unit of red blood cell concentrate that the patient received in December 2021. The regional centre confirmed that the donor's blood was negative for plasmodium blood parasites. Even though the screening test is not performed routinely in blood donations and asymptomatic persons who have visited endemic areas are excluded from donating blood for 6 months after leaving the endemic area [9], in our case, a blood test was performed a posteriori in January 2022 to rule out that the donated blood was infected with *P. falciparum*.

## Hospital-acquired transmission

Even though rare events, cases of hospital-acquired malaria transmission have been described in the scientific literature [10,11]. We checked hospital records of the previous month and identified two patients who were diagnosed with malaria and who had an overlapping period of stay at the hospital. The first case was a patient diagnosed with *P. falciparum* malaria in mid-December 2021. The patient was an individual from the Philippines who arrived in Algeciras on a ship and who was admitted for required treatment of malaria with atovaquone 250 mg and proguanil hydrochloride 100 mg, 1 g every 24 hours. They were finally considered the source case because both (source case and the case presented here) were hospitalised on the same ward, over the same 3 days in December (Figure).

**Figure:**
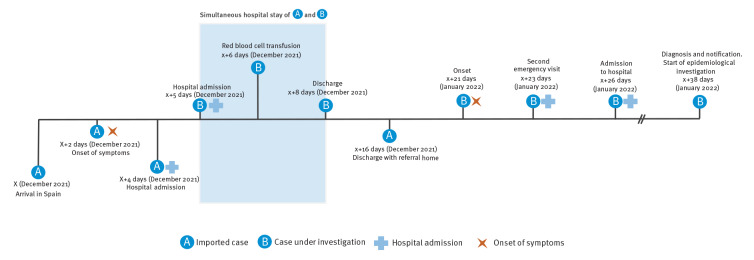
Chronological evolution of the malaria cases, Campo de Gibraltar, Cadiz, Spain, January 2022

Source: Campo de Gibraltar East/West Epidemiology Service, 2022.

The admission floor where both patients stayed for 3 days was identified as a common location, which made it necessary to further investigate hospital-acquired transmission as the possible cause of infection. Two visits were made to the hospital to review the protocol for the preparation and administration of medications by the nursing staff, the handling and disposal of the material, and the use of personal protective equipment. No breeches in procedures were identified.

## Discussion

According to the latest World Health Organization global malaria report in 2021, there were 241 million cases and 627,000 deaths. Six countries accounted more than half of all malaria deaths in 2020 [1]. In the European Union, 99% of cases are laboratory-confirmed and practically all correspond to imported cases, with 99.8% related to international travel [12]. With improvements in the health system and the socioeconomic situation starting in the first half of the 20th century, as well as better vector control, Spain managed to interrupt the transmission of the parasite and declared it eradicated in 1964 [4]. However, in 1978, 1998, 2010, and 2011, non-imported malaria cases were identified in Spain and all were classified as hospital-acquired [10].

Our investigation revealed that the patient shared the admission floor for 3 days with a confirmed case of imported malaria due to infection with *P. falciparum*. The two most recent cases of hospital-acquired malaria in Spain occurred in 2016 and 2018, both cases were attributed to nosocomial transmission and confirmed by a positive molecular analysis as local transmission [11]. In our case, a molecular analysis could not be performed because no samples from both cases were available for comparison. However, having ruled out other routes of transmission, and considering the strong epidemiological link, hospital-acquired parenteral transmission is considered the most likely route, although not demonstrable in this investigation. With no failures identified in the hospital procedures, the quality manager was nevertheless informed of the need to review the protocols and procedures. We believe that the most likely route of transmission is related to the use of medical supplies or contaminated fomites. To avoid in-hospital transmissions, it is essential to follow the protocols for handling medical material, use single-dose medications, use disposable equipment for each patient, and frequent hand washing.

## Conclusions

The identification of cases of hospital-acquired malaria reported in Spain and Europe in recent years makes it essential to maintain and reinforce hygienic measures and patient safety practices. The delay in diagnosis, the presence of the vector in our territory, migration, globalisation and climate change, among other factors, increase the probability that there may be local transmission. No entomological investigation was carried out as vector transmission from local *Anopheles* spp. was considered unlikely, as the time of year is incompatible with the mosquito’s life cycle. Epidemiological and entomological surveillance continues to be fundamental for early detection and if necessary, control measures for malaria cases.
